# Incongruence in Doping Related Attitudes, Beliefs and Opinions in the Context of Discordant Behavioural Data: In Which Measure Do We Trust?

**DOI:** 10.1371/journal.pone.0018804

**Published:** 2011-04-26

**Authors:** Andrea Petróczi, Martina Uvacsek, Tamás Nepusz, Nawed Deshmukh, Iltaf Shah, Eugene V. Aidman, James Barker, Miklós Tóth, Declan P. Naughton

**Affiliations:** 1 School of Life Sciences, Kingston University London, Kingston upon Thames, Surrey, United Kingdom; 2 Department of Psychology, University of Sheffield, Sheffield, United Kingdom; 3 Faculty of Physical Education and Sport Sciences, Semmelweis University, Budapest, Hungary; 4 School of Pharmacy and Chemistry, Kingston University London, Kingston upon Thames, Surrey, United Kingdom; 5 Land Operations Division, Defence Science and Technology Organisation, Edinburgh, Australia; Universidad Europea de Madrid, Spain

## Abstract

**Background:**

Social psychology research on doping and outcome based evaluation of primary anti-doping prevention and intervention programmes have been dominated by self-reports. Having confidence in the validity and reliability of such data is vital.

**Methodology/Principal Findings:**

The sample of 82 athletes from 30 sports (52.4% female, mean age: 21.48±2.86 years) was split into quasi-experimental groups based on i) self-admitted previous experience with prohibited performance enhancing drugs (PED) and ii) the presence of at least one prohibited PED in hair covering up to 6 months prior to data collection. Participants responded to questionnaires assessing a range of social cognitive determinants of doping via self-reports; and completed a modified version of the Brief Implicit Association Test (BIAT) assessing implicit attitudes to doping relative to the acceptable nutritional supplements (NS). Social projection regarding NS was used as control.

PEDs were detected in hair samples from 10 athletes (12% prevalence), none of whom admitted doping use. This group of ‘deniers’ was characterised by a dissociation between explicit (verbal declarations) and implicit (BIAT) responding, while convergence was observed in the ‘clean’ athlete group. This dissociation, if replicated, may act as a cognitive marker of the denier group, with promising applications of the combined explicit-implicit cognitive protocol as a proxy in lieu of biochemical detection methods in social science research. Overall, discrepancies in the relationship between declared doping-related opinion and implicit doping attitudes were observed between the groups, with control measures remaining unaffected. Questionnaire responses showed a pattern consistent with self-reported doping use.

**Conclusions/Significance:**

Following our preliminary work, this study provides further evidence that both self-reports on behaviour and social cognitive measures could be affected by some form of response bias. This can question the validity of self-reports, with reliability remaining unaffected. Triangulation of various assessment methods is recommended.

## Introduction

Epidemiological and social science research assessing social cognitions linked to doping behaviour has been constrained by the almost exclusive use of self-report methodology [1]. Anti-doping prevention programmes are also evaluated via self-reported changes in attitudes and willingness to use doping substances, anabolic steroids in particular [2]–[5]. However, recent research has drawn attention to a potential distorting effect of social desirability observed in self-reported social cognitive measures related to doping [6], [7].

In a recent project, benefitting from a multidisciplinary approach combining social and analytical sciences [8], evidence has emerged that potentially could change the landscape of social science research into doping. We have shown, albeit on a small sample, that taking self-reports at face value could lead to misleading conclusions about the social cognitive processes that underlie doping behaviour. For the first time in social science doping research, our results showed that not only the information on doping behaviour but also self-reported social cognitive measures could also be affected by some form of response bias. Whilst differences in explicit (self-reported) social cognitive measures between user and non-user groups were observed in the expected direction when groups were created from self-report, generally the reverse was evidenced when the user status was based on hair analysis results (i.e. based on the presence of at least one prohibited performance enhancing drug in hair). Implicit measures were consistent with the grouping based on hair analysis. The outcome of this project suggested that respondents may consistently manipulate their answers on all related measures in order to maintain the image they wish to project, although the possibility that this response bias might stem, at least partially, from self-deception (as opposed to strategic responding for impression management) cannot be ruled out.

The strikingly different patterns in self-reports and implicit associations in the context of behavioural data inevitably lead to the question of: Which data should we trust? The self-report methodology has endured a mix of support and criticism in the past. Whilst a plethora of literature suggests that self-report can yield a valid assessment of substance use behaviour, it has also drawn equally strong criticism. For example, the Timeline Follow-Back procedure is one of the widely used methods in clinical and research settings to accrue quantitative retrospective estimates of substance use or risky behaviour covering 7 days to 2 years prior to the interview date [9]. It has demonstrated utility in assessing alcohol, marijuana and tobacco use, as well as sexual behaviour involving taking risks of HIV infection, vis-a-vis face-to-face and over the phone interview or internet application [10]-[12] when covering a shorter period, but produces increasingly less accuracy as the timeframe increases [13]. In a similar vein, alternative questionnaire methods have been developed and reviewed for validity and reliability in identifying substance abuse. These include, but are not limited to, the Lifetime Drug Use History [14], Risk Behavior Assessment of lifetime and recent use of amphetamine [15], Drug Abuse Screening Test [16], the CAGE [17] and Cannabis Use Problems Identification Test [18].

Whilst these tests have provided adequate evidence of validity and reliability, other studies using various biomarkers to validate self-reported behaviour data have put convincing evidence forward for a considerable under-reporting of substance use [19]-[22]. A systematic review [23] revealed that this bias is not limited to socially undesirable behaviours; it also extends to simple measures such as height and weight with a tendency towards over- and under-reporting, respectively [23]. The effect of gender, race, age and contextual contingencies such as drug type and seriousness of the offence on over- and under-reporting substance use has also been investigated [24] with race as the only factor so far demonstrating an effect on admitting drug taking behaviour.

Although laboratory experiments manipulating the conditions from neutral to high demand for socially desirable responding are abundant [25], evaluating the facets of potential response bias in field research is seriously compromised by the absence of available independent objective verification [26]. Therefore, the present project aimed to address this gap by triangulating two behavioural measures and two distinct assessment procedures. Specifically, the current paper repeats the previous analysis [8] on a larger sample to verify our preliminary results and seek further evidence to ascertain whether responses given by the different user groups show a distinct pattern. The importance of this question is underscored by the fact that whilst all of these options distort the data obtained from self-reports, they lead to fundamentally different consequences for the inferences made from these data. Random untruthful answers are likely to lead to statistically non-significant results and potentially low internal consistency in measures. Consistently manipulated answers, regardless if the cause is unconscious or strategic, evidently lead to incorrect, but entirely believable conclusions about doping users vs. non-users.

To further explore issues that emerged from our previous study and were reported as preliminary findings [8], we formulated the following hypotheses.

To examine to what extent self-reported doping use is influenced by strategic responding (response bias), and to what extent the implicit assessment remains unaffected by the same bias, we hypothesise that:

H_1_: self-reports align with self-declared use, and the doping BIAT aligns better with user grouping based on hair analysis;
H_2_: we expect strong divergence between explicit and implicit attitude measures among deniers, and small convergence in the clean athlete group.

Based on a previous study showing domain specificity in the behaviour – social projection relationship where self-declared doping users overestimated the prevalence of doping but not social drugs and vice versa [27], we hypothesise that non-doping related variables are unaffected by the behavioural context.

H_3_: No difference exists in self-reported non-doping variables across doping user groups (including both self-declaration and hair-analysis.

As literature precedence shows gender difference in areas prone to socially desirable responding such as dietary intake [28], [29], it was plausible that the level of honesty/dishonesty about sensitive or discriminating behaviour could be affected by gender. Although findings to date are inconclusive regarding the direction of this effect, females generally find lying less acceptable then males [30]–[32]. A recent study investigating dental hygiene found that admission of insufficient dental hygiene doubled for females when a less intrusive method was used, but increased only by 46% among males [33], indicating not only gender but interaction between gender- and method effect. Therefore, regarding potential gender differences, we hypothesise that

H_4_: a gender effect exists on self-reported data for doping behaviour and social cognitive determinants of doping.

## Methods

The study utilised a mixed method design to afford triangulation between explicit measures through self-reported questionnaire, implicit associations using a computerised test for latency measures, and bioanalysis via hair specimens.

### Participants

We expanded the previously used small sample of 14 [8] to the full sample of 82 male and female athletes using convenience sampling. Inclusion criteria were limited to i) holding registration as an athlete in sport clubs/teams and active current participation in organised sports’ competitions and ii) the ability to provide 3 cm of a head hair sample. No specific athlete group was targeted. Competition levels were ranging from university club to international level.

### Recruitment

After securing ethical approval, athletes were recruited via personal contacts to ensure good rapport and trust between the researchers and participants. Athletes received a small payment (value<10€) as compensation for their time and inconvenience. Details on recruitment, inclusion-exclusion criteria, and sample characteristics are provided in Petroczi et al. [8]. The project was approved by the Kingston University Faculty of Science Research Ethics Committee. Participation was voluntary with implied consenting procedure. The Participant Information Sheet, as well as the first page of the questionnaire clearly stated that the completion and return of the questionnaire and submission of the computerised test was taken as consent. The rationale behind omitting written consent was justified on the basis that given the sensitivity (doping use) ensuring complete anonymity was necessary. Data were linked via computer-generated alphanumerical codes. The data collection with implied consent was approved by the Faculty Research Ethics Committee.

### Measures

#### Behaviour

Independent categorical variables were created by dividing the sample into quasi-experimental groups on the basis of i) self-admitted previous experience with prohibited performance enhancing substances and ii) the presence of at least one prohibited performance enhancing drug in hair covering the last 6 months, depending on hair length. This led to four theoretically possible groups as presented in Table 1 along with basic demographic information. Known groups were formed based on self-reports (we refer to this as ‘self-report doping scenario’) or hair analysis (we refer to this as ‘hair analysis doping scenario’). For self-report measure of doping behaviour, athletes were asked whether they have ever used a prohibited performance enhancing substance. The combined approaches (self report and hair analysis together) yield a group where participants denied their experience with doping despite the fact that their hair analysis indicated fairly recent use. For the purpose of this study, we used three groups: self-admitted doping use (group A), self-declared clean athletes (group B) and deniers (group C).

**Table 1:** Athlete groups and demographic information based on self-reports and hair analysis.

	Self report: No doping	Self report: Yes doping
	GROUP C	GROUP D^b^
**Hair Positive**	Male: 2, Female: 8	
	Mean age: 19.50±1.354	
	Individual sports: 6, team sports: 3; unspecified^c^: 1	
	Olympic sports: 7/10	

	GROUP B	GROUP A
**Hair Negative** a	Male: 29, Female: 32	Male: 8, Female: 3
	Mean age: 21.75±3.026	Mean age: 21.73±2.240
	Individual sports: 33, team sports: 16; unspecified^c^: 12	Individual sports: 10, team sports: 1
	Olympic sports: 47/61	Olympic sports: 5/11

a For selected performance enhancing drugs.

b Owing to the different timeframe (lifetime vs. last 6 months) and limited scope of the hair analysis we found no athletes in this group.

c Sports that can be either (e.g. rowing).

Self-reported doping behaviour was established from the answers given to the question: *Have you ever used a banned substance*?, with a dichotomous answer option. At the beginning of the questionnaire, clear and concise definitions were given for doping, social drugs and nutritional supplements with exemplars given for all three groups. The social drug category excluded caffeine, alcohol and tobacco.

Hair samples were cut at scalp with a blunt tip scissor and stored in paper envelopes at room temperature until analysis. It is generally accepted that hair analysis is not suitable for detecting single (possibly accidental) exposure [34]. Incorporation of these performance enhancing drugs requires sustained use during the period the hair sample (length) covers.

#### Psychological assessments

Dependent variables included a wide range of self-reports on social cognitive determinants of doping coupled with a modified version of the Brief Implicit Association Test (BIAT) procedure [35] to doping. The doping BIAT pairs performance enhancement related stimuli in doping (*nandrolone, stanozolol, testosterone, amphetamine*) and nutritional supplement (*vitamins, ginseng, garlic, calcium*) target categories with ‘good’ attributes (*peace, joy, love, smile*). The opposite evaluative term (‘bad’: *sick, hell, poison, fail*) was non-focal [8]. Categories, attributes and their stimuli were shown to participants before the test. Upon incorrect answer, a large red X appeared for 400 ms at the bottom of the page prompting participants to correct their answers. Mean latency difference and D-score were calculated according to the scoring algorithm recommended by Greenwald et al. [36]. Latency time difference and D-score <0 indicates relatively stronger associations for nutritional supplements with good attributes; whereas latency difference time and D-score >0 suggests the opposite.

Explicit social cognitive measures were: doping attitude, using the Performance Enhancement Attitude Scale, PEAS [37]; social projection of doping and nutritional supplement (NS) use prevalence; beliefs whether doping should be allowed in competition and opinion on what proportion of athletes would be found guilty if samples taken today would be analysed in 10 years time. The PEAS is a 17-item, unidimensional measure of general doping attitude with good reliability across several studies [37]. The PEAS is scored on a 6-point Liker-type scale indicating levels of agreement with the attitude statements. Response options were anchored at each point ranging from strongly disagree (1) to strongly agree (6). Example items are: “*doping is an unavoidable part of competitive sport*”, “*health problems related to rigorous training and injuries are just as bad as from doping*” and “*there is no difference between drugs, fibreglass poles, and speedy swimsuits that are all used to enhance performance*”. In this study, the Hungarian version of PEAS with previously established psychometric properties was used [27], [37]. Social projection was solicited by using independent single questions asking respondents to estimate the proportion of the general population using nutritional supplements and illicit drugs; as well as the proportion of athletes in their own sport using nutritional supplements and doping. Estimation was given in percentage between 0 (nobody) to 100% (everyone). These, and the other non-standard questions regarding perceived pressure, preferred competitive situation and doping opinion are provided (Table S1) along with their scoring method. Non-doping related dependent variables (social projection of NS and social drug use among members of the general population) were used as control variables.

#### Bioanalysis

Approximately 100 mg untreated head hair, cut at scalp, was screened using ELISA kits for the presence of the most commonly used anabolic steroids (stanozolol, nandrolone and boldenone). Positive samples for anabolic steroids were confirmed and quantified using liquid chromatography-tandem mass spectrometry (LC-MS/MS) methods [8], [38]. Erythropoietin (EPO) was detected using quantifiable ELISA. Hair digestion and analytical methods using LC-MS/MS were developed in house to increase sensitivity and to reduce the amount of hair required [38].

### Data analysis

Group comparisons are first performed by factorial ANOVA to allow the testing of main and interaction effects. Owing to the unequal, and in some cases too small, group sizes, main effects were then confirmed by non-parametric (Mann-Whitney) tests. Owing to the small sample size for ‘user’ groups, inferential statistics should be read with caution. Means and standard deviations are provided for all dependent variables. Relationships were tested using the chi-square test for independent variables and correlation coefficients (Pearson's r and Kendall's tau) for dependent variables. Effect size and minimum required sample size for statistical significance were calculated using G*Power 3.1 software. Interaction terms were calculated by multiplying the z-scores for the two continuous explicit and implicit attitude measures. The BIAT effect was shown by latency difference (raw scores in ms) and D-scores [36] with negative values representing a longer latency time on the *good + doping* task (vs. *good + nutritional supplement* task). The doping BIAT task was run on a standard desktop computer (AMD Athlon^TM^ 64X2 Dual Core Processor 4400+) under Windows XP operating system, using a bespoke test developed in-house. Response options were assigned to keyboard letters. Statistical analyses were performed using PASW Statistics v18.

## Results

The mean age of the samples was 21.48±2.86 years (range 18–30). Gender distribution of the sample was close to equal (52.4% female). Athletes in the sample represented 30 sports with track & field: 14, triathlon: 7, gymnastics: 7 and soccer: 6 appearing more than five times. Competitive levels ranged from university to national level. Scale reliability (Cronbach alpha) for the PEAS in this study was .806.

### Prevalence rate of the use of performance enhancing substances and nutritional supplements

Nutritional supplement use was reported by 60%, whereas those having personal experience with doping constituted 13.4% of the athletes. Admitted doping appears to be independent of self-reports on NS or social drug use. Eight hair samples were positive for stanozolol and two for EPO, giving a 12% prevalence rate for prohibited performance enhancing substance use in the sample. Detected Stanozolol levels in hair are reported in Petroczi et al., 2010 and Deshmukh et al., 2010. EPO levels were 13.35 pg/mg and 12.53 pg/mg for the two positives. Frequencies by gender are shown in Table 2. Interestingly, more males admitted having experience with doping than females (20.5% vs. 7.0%, respectively) with a reversed pattern for positive hair analysis (5.3% vs. 18.6%, respectively).

**Table 2:** Self-reported use of nutritional supplements and doping in comparison to prevalence of Stanozolol/EPO use based on detection in hair in the sample by gender (presented as frequencies).

	Use	Male	Female	Total^a^
Nutritional supplements self-report	Yes	28	21	49
	No	11	22	33
Doping self-report	Yes	8	3	11
	No	31	40	71
Stanozolol or EPO detected in hair^b^		2	8	10

a one reported prohibited performance enhancement use for medical reason with TUE.

b one Stanozolol level was below the level of quantification (0.5 pg/mg of hair), Deshmukh et al., 2010.

None of the athletes who returned positive hair samples admitted doping use. Conversely, no self-admitted doping was confirmed by current hair sample tests (Table 1). Owing to the evident uncertainty (i.e. whether the mismatch between self-reports and analytical results were due to false reporting or limits in time and/or scope of the hair analysis) around this latter group (group A in Table 1), these athletes were excluded from further analyses but their results are provided in Tables S2 and S3 to inform future research.

### Social cognitive factors

Social projections for NS, doping and social drug use showed a positive, statistically significant but relatively weak relationship between fellow athletes using prohibited performance enhancing drugs and NS (r = .385, p<.001) and social drugs by the general population (r = .231, p = .036).

Based on self-declared doping behavior, an interaction effect between user group and gender was only found for social projection of doping (F = 4.454, p = .038) and NS (F = 4.379, p = .040) use by fellow athletes. No gender difference was evidenced for any of the outcome variables except the pressure to use banned substances, where the t-test showed a statistically significant difference (t(57.67) =  −2.093, p = .041), but the non-parametric test failed to confirm this (p = .071). On the scale of 100% representing maximum pressure, the estimation given by males was higher (18.46%, SD = 28.40 vs. 7.72%, SD = 15.58). Gender difference or interaction effect was not detected in comparing ‘clean’ athletes and ‘deniers’ within the self-declared clear group.

A statistically significant difference was found between admitted doping users and non-users in explicit attitude (p<.001) and social projection of doping use (p = .024), with a borderline significance for pressure to use doping (p = .062). As expected, those who admitted having personal experience with doping exhibited more of a lenient attitude (shown by higher PEAS score) towards doping and gave higher estimated proportions of doping users among athletes and reported higher perceived pressure to use doping. Means, SDs and test statistics are provided in Table 3. These findings are in keeping with literature precedents.

**Table 3:** Groups by self-declared doping behaviour (means, SDs and test statistics for main effects).

Dependent variable	Doping use	Mean ± SD	Mann-Whitney U significance (p)
**Explicit doping attitude (PEAS)**	Yes	48.00±12.24	
	No	34.04±8.12	p<.001
**Perceived pressure to use doping**	Yes	28.64±35.58	
	No	10.38±19.78	p = .062^a^
**Fellow athletes use doping**	Yes	35.45±27.29	
	No	16.97±19.08	p = .024
**Fellow athletes use supplements**	Yes	62.18±31.22	
	No	59.49±24.73	p = .682
**General public use supplements**	Yes	35.73±19.21	
	No	35.30±21.29	p = .859
**General public use social drugs**	Yes	54.36±21.36	
	No	41.59±20.52	p = .067
**BIAT doping (latency in ms)**	Yes	−171.90±223.51	
	No	−92.31±156.91	p = .168
**BIAT doping (D score)**	Yes	−0.280	
	No	−0.249	p = .649

a t-test showed significant difference (t(57.67)  = −2.093, p = .04; equal variances not assumed).

### Triangulating explicit and implicit measures with objective behavioural data

**Figure 1:**
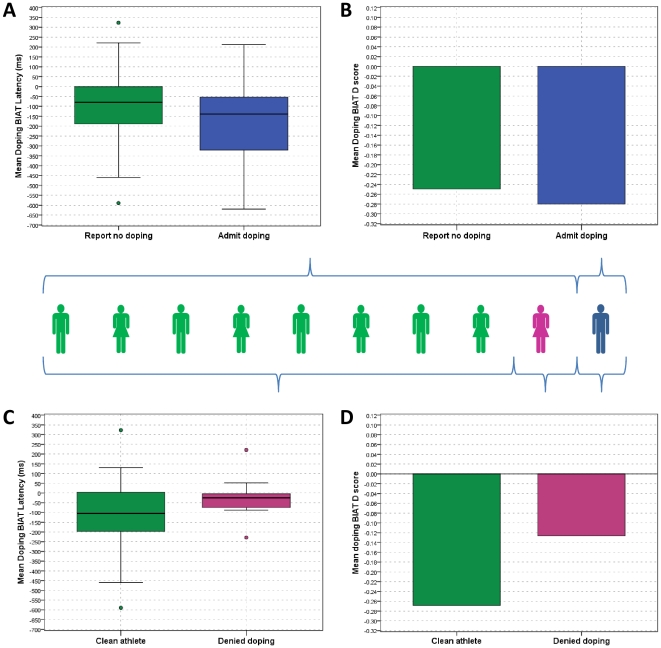
Implicit doping associations by user groups. ‘Persons’ in the middle represents 100% of the sample with persons in green representing clean athletes (n = 61); blue depicts self-reported doping (n = 11); pink shows the proportion of athletes who denied doping use (n = 10), hence would be classified as non-user according to self-reports. Panels are: (A) latency measure on the doping BIAT task by self-reported user groups; (B) D scores of the doping BIAT task by self reported user groups; (C) latency measure on the doping BIAT task based on hair analysis; (D) D scores of the doping BIAT task by self reported user groups. Circles in panels A and C represents outliers.

Adjusting the criteria for establishing user groups with hair analysis results, a new group emerged within self-declared clean athletes, namely those who denied being involved in doping practices yet the drug was present in their hair. Analysis comparing clean athletes and deniers yield, yet again, a very different picture than the one that was obtained based on self-reports. For comparison, we provide means, SDs and test statistics of the main user group effect for the same set of dependent variables in Table 4. Owing to the uncertainty around the self-admitted doping users, these athletes have been excluded. Figure 1 illustrates the effect of the behavioural information on observed differences in latency measures and D-scores derived from the implicit association task (BIAT). In panels A and B, user groups were based on self reported information on doping. As revealed by subsequent hair analysis, a proportion of athletes (shown in pink) among those who were classified as non-users based on self-declaration (shown in green) were, in fact, users. Consequently, the results of self-declared non-users are confounded by the not-insignificant cohort of athletes who used doping but denied this key information on the self-reported questionnaire.

**Table 4:** Means, SD and test statistics in the hair analysis doping behaviour scenario.

Dependent variable	Doping use	Mean ± SD	Mann-Whitney U significance (p)
**Explicit doping attitude (PEAS)**	Deny	30.78±6.85	
	Clean	34.55±8.23	p = .221
**Perceived pressure to use doping** a	Deny	1.00±3.16	
	Clean	11.92±20.92	p = .038
**Fellow athletes use doping**	Deny	13.60±14.59	
	Clean	17.52±19.77	p = .588
**Fellow athletes use supplements**	Deny	56.20±24.70	
	Clean	60.02±24.90	p = .589
**General public use supplements**	Deny	35.00±25.93	
	Clean	35.34±20.69	p = .784
**General public use social drugs**	Deny	44.70±24.98	
	Clean	41.08±19.90	p = .522
**BIAT doping (latency in ms)**	Deny	−21.23±119.90	
	Clean	−103.34±159.91	p = .127
**BIAT doping (D score)**	Deny	−0.126	
	Clean	−0.269	p = .221

a t-test showed significant difference (t(68.96)  = 3.818, p<.001, equal variances not assumed).

Once a new grouping was used, it was notable that the ‘denier’ group performed quite differently on the BIAT, resulting in a smaller latency difference and diminishing IAT-effect (Figure 1C and 1D). In practical terms, those athletes who denied doping use found the *good + doping* pairing less difficult (shown by decreased latency). The same pattern holds for the D-scores, which are derived from latency measures, but after having taken the individual variations in cognitive ability into account. Latency measures and D-scores are detailed in Tables 3 and 4. The lack of significance is owing to the relatively large individual variations combined with the small sample size. The effect size for comparing ‘clean’ athletes and ‘deniers’ was d = 0.581 which would require at least 48 athletes in each group to reach statistical significance. The observed difference, however, is notable and it offers a valuable avenue for future research.

As the means indicate in Table 4, athletes who denied doping contrary to the evidence in their hair samples exhibited less lenient attitudes toward doping. These results support our previous observation that athletes who deny their doping use behaviour gave answers on social cognitive measures that are consistent with a typical non-user. In other word, they are ‘faking good’ in a consistent manner. These results are not surprising, considering that the outcome measures were exclusively based on self-declarations. The picture, however, has changed for the implicit associations. Albeit the differences in latency or D-scores did not reach statistical significance, the sample means suggest that performance on the BIAT was revealing for deniers. That is, latency measures and D-scores set deniers apart from clean and self-admitted users, but did not differentiate between the latter two groups. There are several plausible explanations for fact that the IAT did not differentiate between admitted doping users and self-reported clean athletes (Figures 1A and 1B). First of all, the ‘clean’ athletes' group level measure was confounded by those who denied doping, pulling the mean latency and related D score toward a more neutral position. Secondly, the self-reported doping user is a dubious group membership.

In order to find further evidence that triangulating self-reports and implicit assessments could be a rewarding approach to identify dishonest participants, we calculated and compared the interaction terms between these two measures. The interaction effect (Table 5) showed moderate divergence in the denier group and slight convergence among clean athletes. These results are in keeping with the hypothesis that discrepancies between explicit and implicit measures are greater in groups that have ‘something to hide’ by societal standards.

**Table 5:** Interaction between measured variables (mean, minimum and maximum, respectively).

	Clean athletes	Deniers
Explicit attitude x Implicit association	.2331	−.1734
Implicit association x Pressure	.0782	−.1724
Implicit association x Social projection	.0078	.2928
Explicit attitude x Social projection	.0056	.1682
Social projection x Pressure	.1795	.1547
Explicit attitude x Pressure	.1098	.2966

Finally, we looked at information on preferred competitive situations, beliefs about the necessity of doping to win, opinion regarding the prohibition of doping for elite and all athletes and a projection of the proportion of athletes who would be found positive if samples taken today would be analysed in 10 years was also looked at in the context of behavioural data. The majority of the athletes (89%) indicated that they would prefer to compete in a doping free environment. The remaining 11% opted for a scenario in which both players use doping. The fact that no athletes opted for a unilateral use of doping suggests that there is a proportion of athletes who might be more motivated about enhancing performance in general (including using prohibited means) than gaining competitive advantage against opponents. Congruent with previous findings, 45% of those who admitted doping expressed a preference for a situation with mutual doping use, followed by 10% of those denied doping and 6% of the clean athletes. Interestingly, 62% of all respondents believed that athletes use performance enhancing substances in training and competition. As expected, athletes who denied doping use consistently expressed opinions regarding doping use, necessity and status similar to or even more rigid than those of clean athletes. Detailed analysis is shown in Table 6.

**Table 6:** Doping related opinion by user groups (% is the proportion within the respective group, rounded to the closest full number).

		Clean athlete	Denied doping
Questions	Answer options	n = 61	n = 10
Perceived doping use	Training and competition	38 (62%)	4 (40%)
	Training only	11 (18%)	0
	Competition only	4 (7%)	3 (30%)
	Not used	8 (13%)	3 (30%)
Possible to win without doping?	Yes	41 (68%)	8 (80%)
	No	10 (16%)	2 (20%)
	Do not know	10 (16%)	0
Legalising doping for top level athletes	Yes, without restrictions	0	0
	Yes, but with restrictions	10 (16%)	0
	Absolutely not	51 (84%)	10 (100%)
Legalising doping for all athletes	Yes, without restrictions	0	0
	Yes, but with restrictions	14 (23%)	2 (20%)
	Absolutely not	47 (77%)	8 (80%)
		**n** = **58**	**n** = **9**
Proportion of athletes ‘clean’ today but ‘guilty’ in 10 years	None	0	0
	A few	8 (14%)	3 (33%)
	A solid minority	7 (12%)	2 (22%)
	Half	18 (31%)	1 (11%)
	Majority	24 (41%)	3 (33%)
	All of them	1 (2%)	0

## Discussion

The aim of this study was to expand on and re-examine our previous findings suggesting that self-report regarding doping is contaminated by a response bias whereas the implicit doping-related associations are less affected by it. A pattern similar to that described in Petroczi et al [8] was observed in the extended data set, thus confirming our preliminary findings. In terms of the extremes of the user spectrum (clean athletes and those who denied doping but with hair analysis showing the presence of a performance enhancing drug), self-reports aligned well with self-declared use, whereas the implicit association test (doping BIAT) results aligned better with user grouping based on hair analysis. We further hypothesized a strong divergence between explicit and implicit attitude measures among deniers; and small convergence in the clean athlete group. These assumptions have been verified although perhaps owing to the small sample and relatively large variance, the strength of divergence was not high.

In addition to confirming the previously found discrepancies in self-reports as a function of group identification (i.e. whether it was based on self-reports or hair analysis), we have found that whilst males dominated the self-declared doping user group, the number of females were disproportionately high among deniers, suggesting some gender effect on both self-admissions and denials. Intriguingly, gender effect was not found on any of the dependent variables, indicating that despite the observed pattern in the discrepancy between self-reported and verified behaviour, gender had no systematic effect on self-reported data for social cognitive determinants of doping. We have also found further evidence that distorted social projection (false consensus) is only present for the doping related sensitive issues, not for general population social drug use or NS. Non-doping related variables are unaffected.

The similar performance on the implicit association test (BIAT) by clean athletes and self-admitted users is somewhat unexpected. Further investigation is required to untangle this phenomenon, but one possible explanation is that the two groups hold dissimilar beliefs about and attitude toward both NS and doping substances, resulting in different latency on both pairings but similar latency difference and D score. As the implicit test required sorting words into categories where either doping or supplements were, as targets, paired with the ‘good’ attribute category difference in the IAT effect can only be expected if members of one group hold a different view about the pair of interest (e.g. *good + doping*) and thus perform the task with relative ease in comparison to the other group, but all athletes hold similar views and perform similarly on the comparing task (e.g. *good + nutritional supplements*). In a theoretical case where implicit associations differ on both targets, it is possible to derive a similar IAT effect score with very different performance on the paired tasks. Group mean latency times on each task pairing appear to support this assumption. Self-admitted doping users performed significantly slower on both task pairs than their self-declared clean counterparts (data not shown). Our implicit association tasks contrasted performance enhancing substances and nutritional supplements. Both are often used by athletes with the only difference being in their legal status (i.e. prohibited in sport competition or not). Future studies looking into contrasting deliberately distorted responses on explicit measures and implicit association among ‘deniers’ should incorporate alternative implicit association tasks independent of potential (non-prohibited) performance enhancements. However, our results suggest, with considerable confidence, that the ‘denier’ group is characterised by a pattern of dissociation between explicit and implicit responding. This dissociation is, in fact, likely to be a cognitive marker for this group, which may lead to a promising application of the combined explicit-implicit cognitive protocol used in this study as a proxy for the less readily available biochemical detection methods for large scale social science research on doping.

General population NS and social drug use estimates were used as controls (i.e. non-sensitive issue). However, whilst it served its main purpose, estimations also showed an interesting pattern, hinting that response bias observed in the socially sensitive domain of doping might be linked to the perception of doping as ‘drug’ (as oppose to functional aid). This is consistent with recent theorising that doping attitudes and behaviours may depend on how doping is represented in the athlete's mind - as a drug use (doping as an illicit behaviour) or as an ergogenic aid (doping as functional use) [39], [40]. Investigating contextual influences, Smith et al. [41] concluded that athletes' views on doping were first and foremost influenced by the ‘legality’ of the substance, then on performance. In this, athletes interviewed considered prohibited performance enhancing substances as cheating but acceptable enhancers as essential. This conclusion maps neatly to our results on nutritional supplements and doping agents but also calls for further investigation as our results on the implicit association task might have been affected by the target stimuli [42]. Following Payne and Gawronski's recommendation [43], future doping research should aim to understand the roots of performance differences in the doping-related explicit and implicit assessment protocols in the context of external validation criteria (e.g., behavioural and bio- markers).

### Limitations

Limitations associated with this study include convenience sampling and partly the study design itself. With regard to the prevalence rate, available prevalence statistics are either based on adverse analytical findings or some type of self-reports, which makes direct comparison with our results impossible. Furthermore, owing to the different timeframes and the limited scope of the hair analysis, the extent of a possible overlap between the two subsets of positives is unknown, hence the two prevalence rates (13.4% and 12%) cannot be combined for a lifetime prevalence estimate. We have opened up the self-reports to any prohibited performance enhancing substances in order to obtain a reasonable sample size for self-declared doping users and although this has led to the omission of Group A from part of the analysis, it did not have an effect on the key group (deniers). Future studies may recruit strategically and restrict both the timeframe and the substance list for a complete overlap between self-reports and bioanalysis. Future investigations could also benefit from using more detailed questions and alternative indirect methods regarding doping use. Longer hair samples (if available) may be sectioned by time (e.g. 2-week or 1-month segments) to potentially identify those who may have just started to use doping as this may influence their willingness to admit this behaviour. However, this approach would also require validation studies showing the time for which the drug in question remains in the hair and drug mobility along the hair shaft *in vivo*.

### Conclusion

The results of this study draw attention to the discrepancy in doping related explicit attitudes, beliefs and opinions in the context of behavioural data; and to the unique, but perhaps revealing patterns observed in implicit cognition. The most important contribution that our results can add to drug use research is the observed distinct patterns of explicit and implicit responding among self-declared doping users and deniers which may lead to significant advances in both detection and treatment interventions for these groups. Our findings question the validity of self-reports which may have significant implications in interpreting previous and future doping research. A combination of self-report and implicit cognitive measures seems to hold the strongest promise for future doping research. It is this combination that is likely to produce, with attendant methodology refinements, robust cognitive markers of denial. Objective verification using biomarkers or chemical analysis may not be a feasible approach in all social science research. However, our results suggest that triangulating results obtained on the same or related constructs but using different methodologies could be a cost-effective avenue.

Hence, further research into the methods of combining self-report methodology, with indirect, implicit methods is warranted. Assuming that social desirability has a root in contextual contingencies, research among different user groups could be beneficial. Doping social science research, particularly quantitative research, is seriously lacking in studies using samples drawn from athletes banned from competition owing to doping offences and longitudinal research. Research in this field would benefit from looking beyond doping and having a greater use of direct and indirect methods from social psychology, particularly those used successfully in substance use and addiction research. Incorporating implicit social cognition is one promising avenue for doping social science research. Although it is still debated whether implicit social cognitions reveals something about the individual or the individual's environment, implicit social cognition research is among the thriving areas in social psychology. Doping research, owing to the unique nature of doping (i.e. being positioned between illicit behaviour and functional use of ergogenic aids) provides an excellent testing field for developing a better understanding of the explicit and implicit social cognition and the environment.

## Supporting Information

Table S1 **Non-standard questions used in the athlete survey.**
(DOC)

Table S2 **Means and SD for the dependent variables in the self-declared doping user group (Group A).**
(DOC)

Table S3 **Doping related opinion (% is the proportion within the respective group, rounded to the closest full number) in the self-declared doping user group (Group A).**
(DOC)
