# Supplementary material for: Incongruence in Doping Related Attitudes, Beliefs and Opinions in the Context of Discordant Behavioural Data: In Which Measure Do We Trust?
Source: PLoS One. 2011 Apr 26;6(4):e18804. doi: 10.1371/journal.pone.0018804 (PMC3082532; doi:10.1371/journal.pone.0018804)
Supplement: Table S1 — Non-standard questions used in the athlete survey. (DOC) [file pone.0018804.s001.doc]

**Table S1. Non-standard questions used in the athlete survey.**

|  | **Question** | | **Answer option** |
| --- | --- | --- | --- |
| 1 | What % of the general population do you think use nutritional supplements regularly? | | |
|  | | | % between 0 and 100 |
| 2 | What % of the general population you think has used a social drug? | | |
|  | | | % between 0 and 100 |
| 3 | What % of others in your sport is using nutritional supplements? | | |
|  | | | % between 0 and 100 |
| 4 | What % of others in your sport has used a banned substance? | | |
|  | | | % between 0 and 100 |
| 5 | Have you ever used a banned substance? | | |
|  | | | Yes/No |
| 6 | If performance enhancing drugs were effective and increased the possibility of winning, which situation would you rather compete in? | | |
|  |  | a | You use drugs and your opponent does not |
|  |  | b | Your opponent uses drugs and you do not |
|  |  | c | You use drugs, so does your opponent |
|  |  | d | No one uses drugs |
| 7 | Do you think that most high performance athletes … | | |
|  |  | a | use performance enhancing substances in training and competition |
| 8 |  | b | use performance enhancing substances in training only |
| 9 |  | c | use performance enhancing substances in competition only |
| 10 |  | d | do not use performance enhancing substances |
| 11 | How much pressure do you feel to use banned substances? If zero % means no pressure at all and 100% represents maximum pressure, what is the percentage that describes the pressure you feel? | | |
|  | | | % between 0 and 100 |
| 12 | Do you believe that performance-enhancing drugs/methods should be allowed for all / top level athletes? (used as separate questions) | | |
|  |  | a | Yes, without restrictions |
|  |  | b | Yes, but with restrictions |
|  |  | c | Absolutely not |
| 13 | Do you believe that it is possible to win in high level sport competitions without doping? | | |
|  | | | Yes/No |
| 14 | How many “clean” champions of today do you think will be found guilty of doping violations when their samples get unfrozen and analysed in 10 years time? | | |
|  |  | a | none |
|  |  | b | a few |
|  |  | c | a solid minority |
|  |  | d | half |
|  |  | e | majority |
|  |  | f | all of them |
