# Supplementary material for: Incongruence in Doping Related Attitudes, Beliefs and Opinions in the Context of Discordant Behavioural Data: In Which Measure Do We Trust?
Source: PLoS One. 2011 Apr 26;6(4):e18804. doi: 10.1371/journal.pone.0018804 (PMC3082532; doi:10.1371/journal.pone.0018804)
Supplement: Table S2 — Means and SD for the dependent variables in the self-declared doping user group (Group A). (DOC) [file pone.0018804.s002.doc]

**Table S2. Means and SD for the dependent variables in the self-declared doping user group (Group A).**

| **Dependent variable** | **Mean ± SD** |
| --- | --- |
| Explicit doping attitude (PEAS) | 48.00 ± 12.24 |
|  |  |
| Perceived pressure to use doping | 28.64 ± 35.57 |
|  |  |
| Fellow athletes use doping | 35.45 ± 27.43 |
|  |  |
| Fellow athletes use supplements | 62.18 ± 31.22 |
|  |  |
| General public use supplements | 35.73 ± 19.20 |
|  |  |
| General public use social drugs | 54.36 ± 21.36 |
|  |  |
| BIAT doping (latency in ms) | -171.90 ± 223.51 |
|  |  |
| BIAT doping (D score) | -0.280 |
|  |  |
