# Supplementary material for: Incongruence in Doping Related Attitudes, Beliefs and Opinions in the Context of Discordant Behavioural Data: In Which Measure Do We Trust?
Source: PLoS One. 2011 Apr 26;6(4):e18804. doi: 10.1371/journal.pone.0018804 (PMC3082532; doi:10.1371/journal.pone.0018804)
Supplement: Table S3 — Doping related opinion (% is the proportion within the respective group, rounded to the closest full number) in the self-declared doping user group (Group A). (DOC) [file pone.0018804.s003.doc]

**Table S3. Doping related opinion (% is the proportion within the respective group, rounded to the closest full number) in the self-declared doping user group (Group A).**

|  | **n = 11** | |
| --- | --- | --- |
| Perceived doping use | Training and competition | 9 (82%) |
|  | Training only | 2 (18%) |
|  | Competition only | 0 |
|  | Not used | 0 |
| Possible to win without doping? | Yes | 4 (36%) |
|  | No | 5 (45%) |
|  | Do not know | 2 (18%) |
| Legalising for top level | Yes, without restrictions | 1 (9%) |
|  | Yes, but with restrictions | 2 (18%) |
|  | Absolutely not | 7 (64%) |
|  | missing | 1 (9%) |
| Legalising for all | Yes, without restrictions | 1 (9%) |
|  | Yes, but with restrictions | 5 (45%) |
|  | Absolutely not | 5 (45%) |
|  | **n = 10** | |
| Proportion of athletes ‘clean’ today but ‘guilty’ in 10 years | None | 0 |
|  | A few | 3 (30%) |
|  | A solid minority | 1 (10%) |
|  | Half | 2 (20%) |
|  | Majority | 4 (40%) |
|  | All of them | 0 |
